# A role for the rare endogenous retrovirus β4 in development of Japanese fancy mice

**DOI:** 10.1038/s42003-020-0781-z

**Published:** 2020-02-04

**Authors:** Akira Tanave, Tsuyoshi Koide

**Affiliations:** 1 Mouse Genomics Resource Laboratory, National Institute of Genetics, 1111 Yata, Mishima, Shizuoka 411-8540 Japan; 2 Laboratory for Mouse Genetic Engineering, RIKEN Center for Biosystems Dynamics Research, 1-3 Yamadaoka, Suita, Osaka 565-0871 Japan; 3 Department of Genetics, SOKENDAI (The Graduate University for Advanced Studies), 1111 Yata, Mishima, Shizuoka 411-8540 Japan

**Keywords:** Molecular evolution, Genomics, Transposition

## Abstract

Two coat-color mutations, *nonagouti*, which changes coat color from wild-type agouti to black, and *piebald*, which induces irregular white spotting, are the characteristics of Japanese fancy mouse strain JF1/Ms. In our *Communications Biology* article, we reported that insertion of a rare type of endogenous retrovirus β4 has caused both coat color mutations. Although there are some reports on the roles of β4 in the mouse genome, further studies on β4 will uncover new features of endogenous retrovirus sequences.

## Abstract (web summary)

In light of their recent finding that insertion of a rare endogenous retrovirus, β4, is the cause of the characteristic coat coloring in agouti and piebald mice, Akira Tanave and Tsuyoshi Koide now discuss the origin and expansion of this element as well as potential roles of β4 in the mouse genome.

## Genetic mutations in coat color genes

Coat color mutation in animals remains one of the most attractive phenomena for both research scientists and the general public. Rudimental genetics of coat color mutations in mice has been reported in historic literature from the Edo period of Japan^1^. Since phenotype is determined by the appearance and the mode of inheritance is relatively simple, various coat color mutations have been identified and documented to date^2^. In the early days of understanding mice genetics, researchers had investigated the coat color mutations as a model of heredity. Many researchers obtained coat color mutations from a stock of fancy mice which was kept by fanciers at that time. This series of mutations is now known as classical mutations.

Laboratory mice have some classical coat color mutations such as *nonagouti* (*a*), *brown* (*Tyrp1*^*b*^), *albino* (*Tyr*^*c*^), *dilution* (*Myo5a*^*d*^), and *piebald* (*Ednrb*^*s*^). Most genetic causes of these mutations have been identified. The *Tyrp1*^*b*^ and *Tyr*^*c*^ mutations caused by point mutations in the protein-coding sequences, would disrupt the protein function and reduce the pigmentation^3,4^. On the other hand, insertional mutations in *a*, *Myo5a*^*d*^, and *Ednrb*^*s*^ alleles have been found to prevent gene transcription and result in hypomorphic phenotype^5,6^. These insertional mutations were caused by insertions of some types of endogenous retrovirus sequence^7^.

In the classical *nonagouti* (*a*) mutation with black coat color, the causal genetic mutation was first identified by Bultman and co-workers in 1994. They reported an insertion of sequence consisted of a 5.4 kb VL30 retroviral sequence containing 5.5 kb unknown sequence in the first intron in *nonagouti* (*a*) allele^8^. Several spontaneous reverse-mutations from nonagouti to agouti coat colors were found to be completely linked with an exclusion of the inserted sequence caused by homologous recombination between the long terminal repeat (LTR) sequences of the VL30 element. Based on these evidences, the cause of *nonagouti* mutation was believed to be the insertion of VL30.

## β4 retroviral insertion in *nonagouti* allele

A quarter century later, we noticed that Japanese wild-derived mouse strain, MSM/Ms (MSM), has an insertion of VL30 in the *agouti* gene even though they exhibit agouti coat color. In order to resolve the inconsistency between the nonagouti phenotype and the insertion of VL30, we re-examined the genetic analyses of the *nonagouti* allele. In the work published in *Communications Biology*^9^, we reported that an insertion of a poorly characterized 9.3 kb endogenous betaretrovirus group 4 (β4, also named as ERVB4, ERV-β4, or MmERV-β4) element, which was previously presented as 5.5 kb unknown sequence, into the VL30 element in the *nonagouti* allele^8^. Similar to other retroviral mutations, the β4 element caused abnormal expression of the *agouti* gene by preventing transcription and interrupting mRNA splicing. Interestingly, a solo-LTR in the *nonagouti* allele developed via homologous recombination between the two β4-LTR sequences has resulted in partial reverse mutation of the coat color to black-and-tan which has yellow coat only on the ventral side. From these results and the precise deletion experiment of β4 by CRISPR/Cas9, β4 was revealed to be the true cause of the *nonagouti* mutation.

As one of the endogenous retroviruses, β4 elements in the mouse genome were first reported by Baillie and co-workers in 2004^10^. They identified a repetitive sequence within the genome of murid rodents as one of the groups of Betaretrovirus including type B and type D retroviruses, such as the mouse mammary tumor virus and *Mus musculus* type D retrovirus, respectively. Apart from that, LTR sequences of the β4 in the *nonagouti* allele had been described as a repetitive sequence in mouse genome^4,6^. There are at least 2000 copies of β4-LTR sequences, a few disrupted or truncated β4 sequences and one possibly intact β4 full-length sequence distributed in the reference mouse genome. However, there were limited reports on the phenotypic mutation caused by the insertion of β4 element. To our understanding, the *piebald* (*s*) mutation was the only case, which was caused by β4 insertion into the first intron of *endothelin receptor type B* gene (*Ednrb*) in Japanese fancy mice^6,11^, until our report on the *nonagouti* mutation.

## Two β4 elements inserted in coat color genes of Japanese fancy mice

**Fig. 1:**
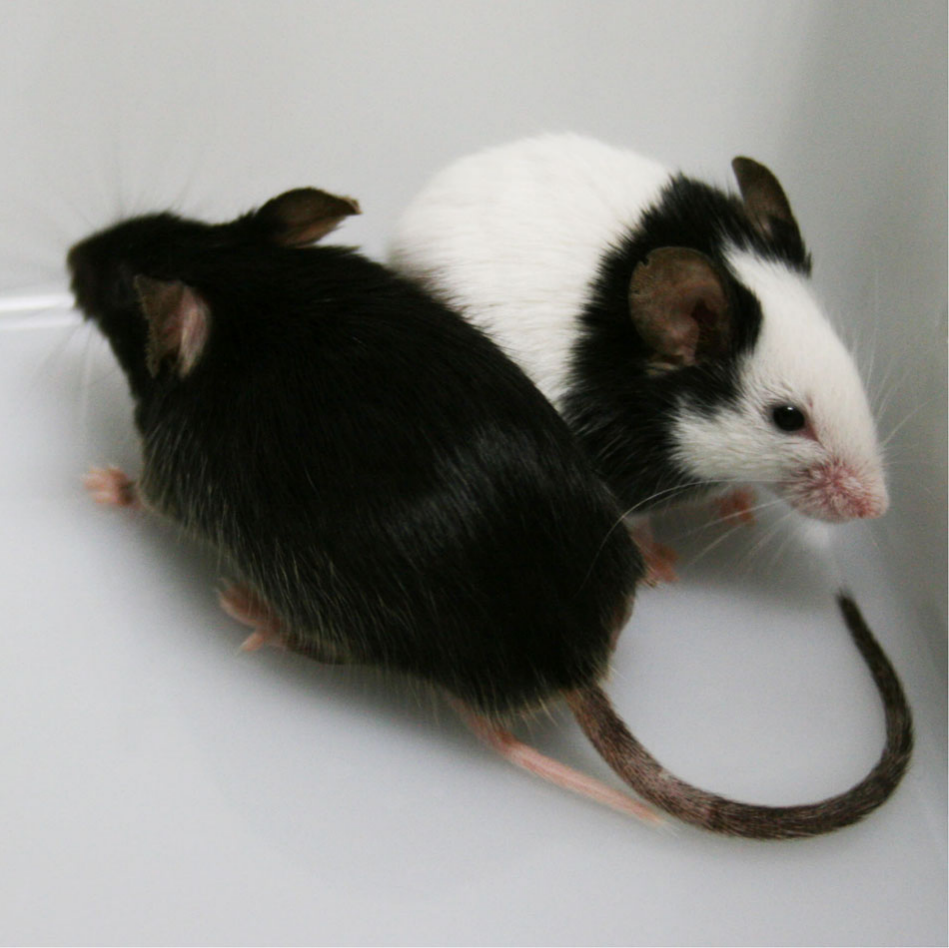
Coat color phenotypes caused by insertion of β4 elements in Japanese fancy mice. Japanese fancy mouse strain, JF1, shows the characteristic combination of nonagouti and piebald phenotypes, in which the β4 elements were inserted in *a* and *Ednrb*^*s*^ alleles (right), respectively. A spontaneous revertant mouse of JF1, JF1-s^+^, which lacks large part of the inserted β4 sequence in the *Ednrb*^*s*^ allele and with restored piebald phenotype (left).

Japanese fancy mouse strain, JF1/Ms (JF1), is known to have two classical coat color mutations, *nonagouti* and *piebald* alleles, leading to characteristic black spots on the white coat, with black eyes^11^. The insertional mutation in the *piebald* allele was identified by Yamada and co-workers in 2006^6^, in which a retroposon-like element was found in the first intron of *Ednrb* gene and had disrupted the normal expressions of the gene. This inserted element was annotated as a truncated type of β4 element in our study^9^. Similar to the case of nonagouti phenotype, spontaneous reverse mutation of the piebald phenotype caused by the homologous recombination between the two β4 LTR sequences was found in JF1 strain, and resulted in nonagouti coat color in a revertant strain JF1-s^+^ (Fig. 1). Moreover, we also found that the insertion of β4 within the VL30 in the *nonagouti* allele has originally occurred in a linage of Japanese fancy mice^9^. All these discoveries indicate that both coat color mutations are insertional mutations caused by retrotransposition of the β4 elements in a specific line of Japanese fancy mice.

This intriguing coincidence implies the possibility of a series of certain mutation processes via retroviral insertion, such as the retroviral expansion in host mouse genome. Although the transposition activity of β4 is still unclear, we and other groups have observed especially high expression level of β4 transcripts in testes^9,12,13^, suggesting a high frequency of germ-line transposition of the β4 elements. The intact viral genes of β4 found in the *nonagouti* allele also indicates sustained transposition activity of the β4 elements^9,10^. Based on these findings, we speculate that the β4 elements were expanded during the domestication process of Japanese fancy mice. In fact, the number of β4 insertions in JF1 mouse genome is about three to seven times higher than in common inbred strains. This high number of β4 insertions is not observed in the closely related wild-derived mouse strain, MSM. These data clearly demonstrate that the insertion of β4 has contributed to the characteristic coat color of Japanese fancy mice.

From an evolutionary perspective, the current known endogenous betaretroviruses including the β4 in the mouse genome are believed to have emerged in the genome of murid rodents at least 20 million years ago^10^. Therefore, some β4 elements that are shared in the homologous loci among related species seems to be orthologs inherited from common ancestors. However, the origin of the β4 element recently inserted in the *nonagouti* allele is unclear except that the element has emerged in the linage of Japanese fancy mice^9^. Although it is highly possible that it comes from a copy that is retrotransposed from another locus, no other identical sequence has been found in the reference mouse genome. Given the intact viral genes, it is also possible that the element has derived from exogenous infection of β4 retrovirus into founder mouse of the Japanese fancy mice.

## The role of β4 elements in mouse genome

Although the role of β4 elements in mouse genome is still unclear, there are several examples indicating a contribution of the β4 to functional genes. For example, the murine-specific C-repeat region of XIST sequence is known to be originally derived from the ERVB4 sequence^14^. This C-repeat sequence interacts with YY1 protein to target XIST RNA to a specific genomic locus^15^. A second example is the *Serpina3a* gene, the first exon of which consists of one part of β4-LTR sequence. Similar to other β4 elements, *Serpina3a* gene is also expressed strongly in testes^16^. The original first exon of *Serpina3a* gene is located on the downstream of the β4-LTR, but its promoter activity is weak and the *Serpina3a* mRNA is transcribed mainly from the β4-LTR sequence. This β4-LTR sequence is shared among the related species and seemed to be inserted before the divergence of *M. musculus*, implying a certain role of this alternative promoter. Therefore, we suggest that *cis*-regulatory elements were introduced in the reproductive system through insertion of β4 in the murine linage.

Beside these significant contributions, high methylation in H3K9me3 and H4K20me3 marks was also found in the β4 sequences^17^, implying a repressive epigenetic state on the β4 similar to the well-known intra-cisternal A-type particle elements. We also observed that a part of the β4 sequences is enriched for CCCTC-binding factor, which regulates three-dimensional chromatin architecture. These characteristics may be related to a role of the β4 in regulating gene expression similar to other LTR retrotransposons^18^, but more detailed analyses are crucial to further elucidate actual functions of β4.

## Concluding remarks

Coat color is an excellent index for studying how mutation alters gene functions that are linked with phenotype. As such, we investigated the insertion of β4 in nonagouti mice and solo-LTR of β4 in black-and-tan mice. There is limited evidence that β4 elements in mouse genome controls gene function. Our findings showed that the retrovirus insertion caused not only a null phenotype but also partial and reversible alternation of the phenotype. Whether β4 had contributed in the domestication and evolution of mouse remains an intriguing issue, and further studies are crucial to understand the roles of β4. Ensuing the studies of coat color mutations, functional studies focused on certain genes containing β4 sequence together with genomic studies using high-throughput sequencing technologies will allow us to uncover how β4 controls gene functions and reveal other roles of β4.
